# Association of HRD Score With Efficacy of Neoadjuvant Short‐Course Radiotherapy Plus Sequential Chemoimmunotherapy in pMMR Locally Advanced Rectal Cancer

**DOI:** 10.1002/cnr2.70708

**Published:** 2026-10-01

**Authors:** Xin Ran, He Xiao, Peng Zhou, Juan He, Mengyuan Si, Xiuqiong Chen, Xiaona Su, Zhuo Chen, Jia Du, Xiaoyan Dai, Xiao Yang, Haode Shen, Zongsheng He, Fan Li, Mengxia Li, Chuan Chen

**Affiliations:** ^1^ Department of Cancer Center Daping Hospital, Army Medical University Chongqing China; ^2^ Armed Police Hospital of Chongqing Chongqing China; ^3^ Department of General Surgery, Colorectal Division Daping Hospital, Army Medical University Chongqing China

**Keywords:** homologous recombination deficiency score, immune checkpoint inhibitors, locally advanced rectal cancer, neoadjuvant radiotherapy, proficient mismatch repair

## Abstract

**Background:**

Homologous recombination deficiency (HRD) has been reported to be associated with increased sensitivity to platinum‐based treatment and radiotherapy in some solid tumors.

**Aim:**

This study investigated the correlation between HRD score and treatment response to neoadjuvant short‐course radiotherapy combined with sequential chemotherapy and tislelizumab in mismatch repair‐proficient (pMMR) locally advanced rectal cancer (LARC).

**Methods and Results:**

Thirty‐two pMMR LARC patients from clinical trial NCT05515796 were enrolled. Pretreatment biopsy samples were analyzed for HRD scores and tumor immune microenvironment (TIME) gene expression profiles. Using receiver operating characteristic (ROC) analysis, patients were stratified into low (0–8), intermediate (9–37), and high (> 37) HRD score groups to compare pathological complete response (pCR) rates. Abundance of immune cells and immune‐related pathways were assessed with MCPcounter and single sample gene set enrichment analysis (ssGSEA) respectively and compared among the three HRD score groups. The entire cohort demonstrated pCR rate of 40.62% (13/32) with a median HRD score of 9 (range: 0–70). ROC analysis showed that HRD score as a continuous variable did not reach statistical significance in distinguishing pCR patients from non‐pCR patients, nor did TMB and HR mutation. However, the proportion of categorized HRD score was significantly different between pCR and non‐pCR groups (low, intermediate, high HRD score: 61.54%, 23.08%, 15.38% vs. 31.58%, 68.42%, 0%, *p* = 0.017). High‐HRD tumors exhibited the highest CD8+ T‐cell abundance followed by low‐HRD and intermediate‐HRD groups.

**Conclusion:**

HRD score may be associated with efficacy of neoadjuvant radioimmunotherapy in pMMR LARC patients, potentially mediated through enhanced CD8+ T‐cell recruitment. These findings support precision patient selection, though mechanistic validation is required.

## 1 Introduction

According to the 2022 GLOBOCAN Global Cancer Statistics report, rectal cancer accounts for approximately 10% of all cancer types and is the second leading cause of cancer‐related deaths worldwide [1]. The primary treatment for early‐stage rectal cancer is radical surgery; however, many cases are diagnosed at a locally advanced stage or with distant metastases. For patients with LARC (T3–4/*N* + M0), the current treatment approach involves multimodal therapy, including neoadjuvant chemoradiotherapy (nCRT) followed by total mesorectal excision (TME) and adjuvant chemotherapy [2]. This strategy has led to improved rates of pCR and overall survival, although pCR is achieved in only 10%–30% of patients [3]. Many clinical studies are now investigating the feasibility of neoadjuvant immunotherapy for LARC [4]. However, only a minority of patients with deficient DNA mismatch repair (dMMR) or high microsatellite instability (MSI‐H) tumors respond to immune checkpoint inhibitors (ICIs), while approximately 85% of colorectal cancer (CRC) patients have so called “cold” tumors especially featured with microsatellite stability (MSS) or low microsatellite instability (MSI‐L) that do not respond well to ICIs [5, 6]. Therefore, it is crucial to explore combination therapies and biomarkers that can activate adapted immunity in “cold” tumors to enhance pCR rates and overall survival.

Radiotherapy (RT) and chemoradiotherapy have been shown to be effective in converting “cold” tumors into “hot” tumors [7, 8]. These treatments induce immunogenic cell death in tumor cells and enhance immune infiltration within the tumor microenvironment (TME), thus establishing a critical foundation for combination therapy with ICIs [9]. Studies using animal models have revealed that different RT fractionation schemes can elicit varying immune‐activating effects. In particular, short‐course RT primarily enhances antitumor immunity compared to long‐course RT [10]. In clinical settings, the VOLTAGE trial indicated that patients with MSS LARC can benefit from nCRT combined with nivolumab. Moreover, patients with high pretreatment tumor tissue PD‐L1 expression and CD8+ T cell infiltration experienced more pronounced tumor regression [11], suggesting that these immune biomarkers may serve as predictive indicators for nCRT. Despite the identification of these potential biomarkers for selecting pMMR LARC patients suitable for neoadjuvant radioimmunotherapy, further investigation into biomarkers based on tumor intrinsic radiosensitivity is essential to enhance the precision and efficacy of this approach.

HRD, a key driver of genomic instability, is prevalent in human cancers. Due to its synthetic lethal properties [12], HRD has emerged as a significant focus in cancer research [13]. Beyond the classical HRD biomarkers, such as germline mutations in BRCA1/2 [14], tumors also exhibit HRD characteristics generated from various factors displaying HRD‐related mutation signatures, copy number variations (CNV), and genomic rearrangements at a genome‐wide level [15]. Among these, genomic scars—such as loss of heterozygosity (LOH), telomeric allelic imbalance (TAI), and large‐scale state transitions (LST)—are independently associated with HRD and can be integrated to define an HRD score to indicate HRD status [16]. Numerous studies have demonstrated that HRD resulting from germline gene mutations can increase the sensitivity of tumor cells to RT and platinum‐based chemotherapy [17, 18]. Furthermore, recent research indicates that patients with HRD‐positive (HRD score > 72) triple‐negative breast cancer (TNBC) experience significant tumor regression following platinum‐based treatment [19]. Additionally, by establishing patient‐derived organoid (PDO) models of rectal cancer to assess the radiosensitivity of native tissue, it was discovered that the HRD status of rectal cancer PDOs determined by mutational signature analysis may significantly influence sensitivity to neoadjuvant RT [20]. Given the high genomic instability closely associated with the HR pathway in LARC [21], further exploration of the impact of HRD score in pMMR LARC on pathologic responsiveness to neoadjuvant radioimmunotherapy is anticipated to yield substantial benefits for this subset of patients during neoadjuvant treatment.

In the present study, the potential of the HRD score as a biomarker for predicting the efficacy of neoadjuvant radioimmunotherapy in LARC was evaluated by using colonoscopy biopsies from a clinical study involving short‐course RT followed by sequential tislelizumab treatment. Additionally, the potential reasons for the varying responses associated with the HRD score were investigated with bulk RNA‐seq data from the point of view of TME.

## 2 Materials and Methods

### 2.1 Patients

This study was conducted on the basis of the clinical trial (NCT05515796). From 2021 to 2023, patients with LARC without dMMR, diagnosed according to the 7th edition of the AJCC staging system at the Third Affiliated Hospital of Army Medical University, received a neoadjuvant treatment regimen consisting of short‐course radiotherapy (SCRT) followed by capecitabine and oxaliplatin (CAPOX) chemotherapy, along with tislelizumab immunotherapy. The aim of this study was to evaluate the safety and efficacy of SCRT combined with chemotherapy and immunotherapy. The primary endpoint was pathological complete response rate. Detailed inclusion and exclusion criteria, as well as clinical design protocols, are available at (clinicaltrials.org, Study Number: NCT05515796). In this marker analysis study, we excluded some patients from the original cohort due to missing clinical data, choosing other treatment options, or poor‐quality biopsy specimens. Based on these criteria, a total of 32 patients with LARC were included in the study (Figure S1). Biopsy samples were obtained during colonoscopy prior to neoadjuvant radioimmunotherapy, and all specimens were evaluated by two professional pathologists. Comprehensive clinical, pathological, and epidemiological data from the 32 patients were collected and collated. Tumor regions with tumor cells > 70% of all cells assessed by Hematoxylin and Eosin staining were selected prior to histopathological molecular analysis of the biopsy specimens.

This study was approved by the Ethics Committee of the institution (Ethical Review of Medical Research [2024] No. 95). All patients signed written informed consent before sample collection. Prior to neoadjuvant combination therapy, tumor biopsy tissues from the patients were collected for assessment of HRD and HR‐associated mutant genes, as well as for bulk RNA sequencing (RNA‐seq) and whole‐exome sequencing (WES). These data were analyzed for correlation with pathological response.

### 2.2 Evaluation of Response

Pathological complete remission (pCR; ypT0N0) or pathological incomplete remission (pIR) was classified based on the presence of residual tumor cells in the surgical specimen. Tumor regression grade (TRG) was assessed according to the modified Dworak and Mandard system [22]. Cases were classified into four categories based on the percentage of residual tumor cells: grade 0—complete tumor regression (0% residual tumor cells); Grade I—subcomplete tumor regression (< 25% residual tumor cells); Grade II—partial tumor regression (25%–50% residual tumor cells); and Grade III—minimal tumor regression (> 50% residual tumor cells). Additionally, samples with 0% residual tumor cells but showing lymph node infiltration (ypT0N+) were classified as Grade II [22].

### 2.3 HRD Score

We entrusted Suzhou Huizhen Medical Laboratory Co. Ltd. to perform HRD scoring on the collected samples. The HRD score was determined based on 40 000 SNPs (with a population frequency close to 50%) from the hg19 genome as probes. Raw reads were processed with cutadapt and fastp software to remove adapters and low‐quality reads. BWA‐MEM2 was used to align the clean reads to the reference genome hg19.3 [23]. The alleleCounter was utilized to calculate the alteration frequency for each SNP. The copy numbers were inferred using ASCAT from the alteration frequencies of SNPs [24]. HRD scores were computed for each sample by considering three metrics: LST, TAI, and LOH [25]. Specifically, LST represents chromosomal breaks > 10 Mb between adjacent regions (high: > 15 in diploid tumors and > 20 in polyploid tumors). TAI refers to the number of subtelomeric regions (extending from the centromere to the telomere) with allelic imbalances (high: > median). LOH indicates the number of LOH regions shorter than the entire chromosome but > 15 Mb (high: > 10).

### 2.4 WES and Tumor Mutation Burden Estimation

Genomic DNA was extracted from biopsies and matched blood samples. Agilent SureSelect Human All Exon V6 was used for library preparation from a total of 200 ng of DNA. Sequencing was performed using the Illumina NovaSeq 6000 for paired‐end 150 bp reads. Clean reads were aligned to the GRCh37/hg19 human reference genome using Burrows–Wheeler Aligner [26]. SNVs and indels were detected by Mutect2 embedded in the Genome Analysis Toolkit GATK (version 4.4.0.0) [27] and annotated using ANNOVAR [28]. Tumor mutation burden (TMB) was calculated using nonsynonymous mutations, including missense, nonsense, and indels, divided by the total size of 33.86 Mb regions covered by WES.

### 2.5 RNA Sequencing and Signature Analysis

The libraries were sequenced on the Illumina NovaSeq 6000 platform, and 150 bp paired‐end reads were generated. The clean reads were mapped to the reference genome using HISAT2 [29]. The read counts for each gene were obtained using HTSeq‐count [30] and transformed into transcripts per million (TPM) for all subsequent analyses. Radiosensitivity index (RSI) was calculated according to the method proposed in the original article [31]. The abundance of infiltrated immune cells was evaluated using the R package MCPcounter (version 1.2.0). Single sample gene set enrichment analysis (ssGSEA) for 29 common tumor‐related pathways was conducted using the R package GSVA (version 1.48.3). Consensus molecular subtype (CMS) classification was evaluated using the R package CMScaller (version 2.0.1). Each sample was assigned a TME subtype based on the ssGSEA enrichment score from 29 common tumor‐related pathways, according to the method reported in the original article [32].

### 2.6 Statistical Analysis

Differences in baseline characteristics between groups were examined using the Chi‐square test or Fisher's exact probability test. The Kruskal–Wallis test was applied to compare continuous variables, such as RSI and TMB, among the three groups categorized based on HRD score. Receiving operating characteristic curves (ROC) curve analysis was used to determine the capacity of HRD score, TMB, and HR mutations in discriminating pCR from non‐pCR.

## 3 Results

### 3.1 Patient Characteristics and Efficacy of Neoadjuvant Combination Therapy

The median age of the patients in the study cohort (32 cases) was 56.5 years (range: 33–75 years), with a high percentage of males (71.88%). According to the American Joint Committee on Cancer (AJCC) 7th edition staging manual, the clinical stage of the 32 patients was as follows: 5 cases of Stage IIA (15.62%), 1 case of Stage IIIA (3.12%), 22 cases of Stage IIIB (68.75%) and 4 cases of Stage IIIC (12.50%). The vast majority (84.38%) of the patients were diagnosed with Stage III LARC. Based on the distance of the tumor from the anus, the tumors were classified as low (0–4 cm, 31.25%), middle (4–8 cm, 68.75%) rectal cancer, with no cases of high rectal cancer (8–12 cm) found. The middle rectal cancer location was the most common (Table 1).

**TABLE 1:** Baseline characteristics of the whole population.

Clinical factor		*n* (%)
Age	> 65	11 (34.38%)
	≤ 65	21 (65.62%)
Sex	Female	9 (28.12%)
	Male	23 (71.88%)
Location	Lower	10 (31.25%)
	Middle	22 (68.75%)
Tumor length	> 4	25 (78.12%)
	≤ 4	7 (21.88%)
cT stage	T2	1 (3.12%)
	T3	29 (90.62%)
	T4	2 (6.25%)
cN stage	N0	5 (15.62%)
	N1	10 (31.25%)
	N2	17 (53.12%)
Clinical stage	IIA	5 (15.62%)
	IIIA	1 (3.12%)
	IIIB	22 (68.75%)
	IIIC	4 (12.50%)
CEA	> 5	15 (46.88%)
	≤ 5	17 (53.12%)
CRM	Negative	17 (53.12%)
	Positive	15 (46.88%)
EMVI	Negative	20 (62.50%)
	Positive	12 (37.50%)
ypT stage	ypT0	13 (40.62%)
	ypT1	2 (6.25%)
	ypT2	4 (12.50%)
	ypT3	12 (37.50%)
	ypT4	1 (3.12%)
ypN stage	ypN0	25 (78.12%)
	ypN1	7 (21.88%)
CMS classification	Unclassified	3 (9.38%)
	CMS1	6 (18.75%)
	CMS2	5 (15.62%)
	CMS3	7 (21.88%)
	CMS4	11 (34.38%)
TME subtype	D	14 (43.75%)
	F	6 (18.75%)
	IE	8 (25.00%)
	IE_F	4 (12.50%)
HR mutation status	Mutation	7 (21.88%)
	Wild type	25 (78.12%)
mrTRG	mrTRG1	9 (28.12%)
	mrTRG2	11 (34.38%)
	mrTRG3	9 (28.12%)
	mrTRG4	3 (9.38%)
cCR	No	13 (40.62%)
	Yes	19 (59.38%)
TRG	TRG0	13 (40.62%)
	TRG1	7 (21.88%)
	TRG2	11 (34.38%)
	TRG3	1 (3.12%)
pCR	Non‐pCR	19 (59.38%)
	pCR	13 (40.62%)

In this study, treatment response assessment results for all enrolled patients before surgery were comprehensively evaluated according to RECIST 1.1 criteria. The percentage of patients who achieved complete clinical remission (cCR) confirmed by endoscopy was 59.38% (19/32). The percentage of pCR (TRG 0) was 40.62% (13/32), indicating a high treatment response rate (Table 1). Frequently mutated genes, such as TP53 (69%), KRAS (59%), PIK3CA (25%), ATRX (9%), ARID1A (9%), and ATR (9%) were observed and were consistent with previous reports. Additionally, a total of seven cases (21.88%) had at least one HR‐related gene mutation in the entire cohort (Figure 1).

**FIGURE 1:**
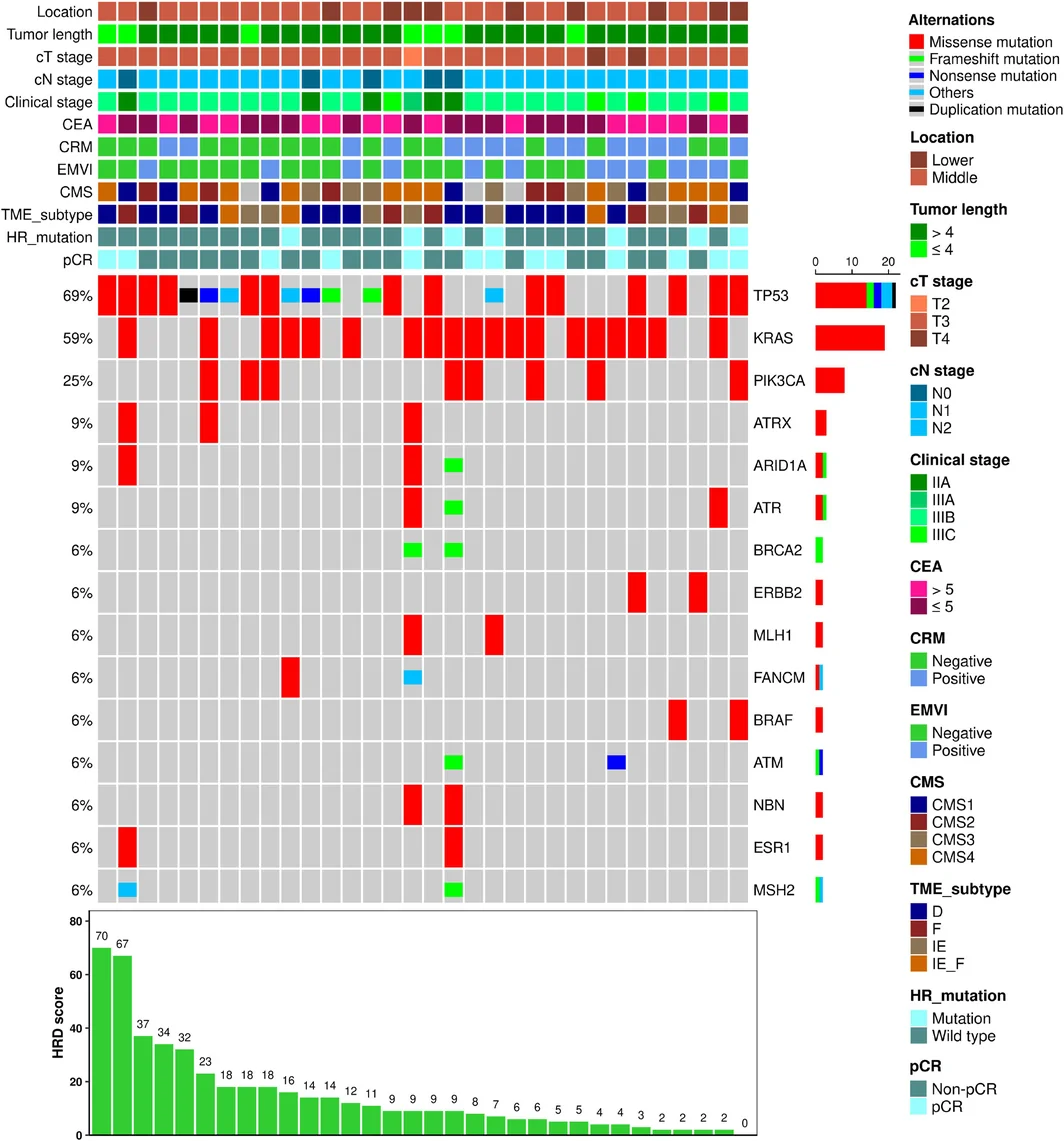
Heatmap of clinical information for 32 patients, mutation frequencies of 15 HRR‐related genes for each patient, and HRD score. Top panel: Heatmap of clinical information for 32 patients, including clinical stage, TNM stage, location, size, CEA, CRM, EMVI, CMS, TME subtype, HR mutation and PCR; Middle panel: Heatmap of mutation frequencies of 15 HRR‐related genes detected by NGS sequencing in 32 patients; Bottom panel: HRD score for 32 patients. CEA: carcinoembryonic antigen, CMS: consensus molecular subtype, CRM: circumferential resection margin, EMVI: extramural vascular invasion, HR: homologous recombination, HRD: homologous recombination deficiency, LARC: locally advanced rectal cancer, PCR: pathological complete response, TIME: tumor immune microenvironment.

### 3.2 Correlation of HRD Score With Pathological Remission

The median HRD score was 9 (range: 0–70) for the entire cohort. Among baseline characteristics, only circumferential resection margin (CRM) status was significantly associated with HRD score. The median HRD score of 6.0 (IQR: 3.5–9.0) in patients with positive CRM was significantly lower than that of 14.0 (IQR: 9.0–18.0) in patients with negative CRM (*p* = 0.027) (Table 2).

**TABLE 2:** Kruskal–Wallis test for the difference in HRD score among clinical factors.

Clinical factor	Subgroups	The whole population (*n* = 32)			Subset of HRD score low and middle groups (*n* = 30)		
		Median (IQR)	*χ* ^2^	*p*	Median (IQR)	*χ* ^2^	*p*
Age	> 65	9.0 (5.0–18.5)	0.006	0.937	9.0 (5.0–18.5)	0.315	0.575
	≤ 65	9.0 (5.0–18.0)			9.0 (4.0–15.0)		
Sex	Female	9.0 (4.0–32.0)	0.099	0.753	5.0 (3.0–16.0)	0.581	0.446
	Male	9.0 (5.5–15.0)			9.0 (5.5–15.0)		
Location	Lower	7.5 (2.7–9.0)	2.035	0.154	7.5 (2.7–9.0)	1.217	0.270
	Middle	11.5 (5.2–18.0)			10.0 (4.7–18.0)		
Tumor length	> 4	8.0 (4.0–16.0)	2.072	0.150	8.0 (4.0–16.0)	0.132	0.717
	≤ 4	9.0 (9.0–42.5)			9.0 (9.0–9.0)		
cTstage	T2–T3	12.0 (6.0–18.0)	1.521	0.218	9.0 (5.5–17.0)	0.745	0.388
	T4	6.0 (4.0–10.0)			6.0 (4.0–10.0)		
cNstage	N0	11.0 (9.0–14.0)	1.310	0.252	10.0 (9.0–11.7)	0.495	0.482
	N1–N2	8.0 (4.0–18.0)			7.5 (4.0–17.5)		
cStage	IIA–IIIA	10.0 (9.0–13.2)	1.134	0.287	9.0 (9.0–11.0)	0.487	0.485
	IIIB–IIIC	7.5 (4.0–18.0)			7.0 (4.0–18.0)		
CEA	> 5	9.0 (3.5–16.0)	0.023	0.880	9.0 (3.2–14.0)	0.053	0.819
	≤ 5	9.0 (5.0–18.0)			8.5 (5.0–16.5)		
CRM	Negative	14.0 (9.0–18.0)	4.905	0.027	14.0 (7.5–18.0)	3.276	0.070
	Positive	6.0 (3.5–9.0)			6.0 (3.5–9.0)		
EMVI	Negative	12.5 (6.7–19.2)	3.732	0.053	10.0 (6.2–17.5)	2.536	0.111
	Positive	5.0 (2.7–9.7)			5.0 (2.7–9.7)		
CMS	CMS1	13.5 (4.5–30.0)	1.546	0.672	9.0 (3.0–18.0)	1.847	0.605
	CMS2	14.0 (6.0–23.0)			14.0 (6.0–23.0)		
	CMS3	7.0 (4.5–11.5)			7.0 (4.5–11.5)		
	CMS4	9.0 (3.0–17.0)			9.0 (2.5–14.2)		
TME subtype	D	10.5 (6.0–20.7)	1.815	0.612	9.0 (6.0–14.0)	1.409	0.703
	F	9.0 (4.5–26.2)			9.0 (3.0–9.0)		
	IE	8.0 (2.0–12.7)			8.0 (2.0–12.7)		
	IE_F	10.0 (3.5–16.5)			10.0 (3.5–16.5)		
HR mutation	Mutation	7.0 (3.0–9.0)	2.486	0.115	7.0 (3.0–9.0)	1.830	0.176
	Wild type	11.0 (5.0–18.0)			9.0 (5.0–18.0)		
mrTRG	mrTRG1	9.0 (2.0–18.0)	3.261	0.353	7.0 (2.0–9.0)	5.439	0.142
	mrTRG2	14.0 (7.5–17.0)			14.0 (7.5–17.0)		
	mrTRG3	5.0 (4.0–9.0)			5.0 (4.0–9.0)		
	mrTRG4	18.0 (12.0–20.5)			18.0 (12.0–20.5)		
cCR	NO	9.0 (4.0–18.0)	0.013	0.908	9.0 (4.0–18.0)	0.176	0.675
	YES	9.0 (5.5–17.0)			9.0 (5.0–14.0)		
pCR	Non‐pCR	11.0 (5.5–18.0)	0.925	0.336	11.0 (5.5–18.0)	3.608	0.057
	pCR	7.0 (4.0–14.0)			6.0 (3.0–8.5)		

In the entire cohort, the Kruskal–Wallis test showed no significant difference in HRD score between the pCR and non‐pCR groups (Figure S2A). ROC analysis also showed that HRD score did not reach statistical significance in distinguishing pCR from non‐pCR, nor did TMB and HR mutations (Figure S2B). However, it was noted that the two cases with the highest HRD scores (70 and 67) achieved pCR. After rigorous analysis of the remaining 30 cases, it was observed that although the Kruskal–Wallis test revealed the difference in HRD score between the pCR and non‐pCR groups had borderline statistical significance (median: 6.0 IQR: 3.0–8.5 vs. 11.0 IQR: 5.5–18.0, *p* = 0.057) (Table 2), only the HRD score reached statistical significance in distinguishing pCR from non‐pCR in ROC analysis (AUC = 0.710, 95% CI: 0.520–0.901) (Figure 2A).

**FIGURE 2:**
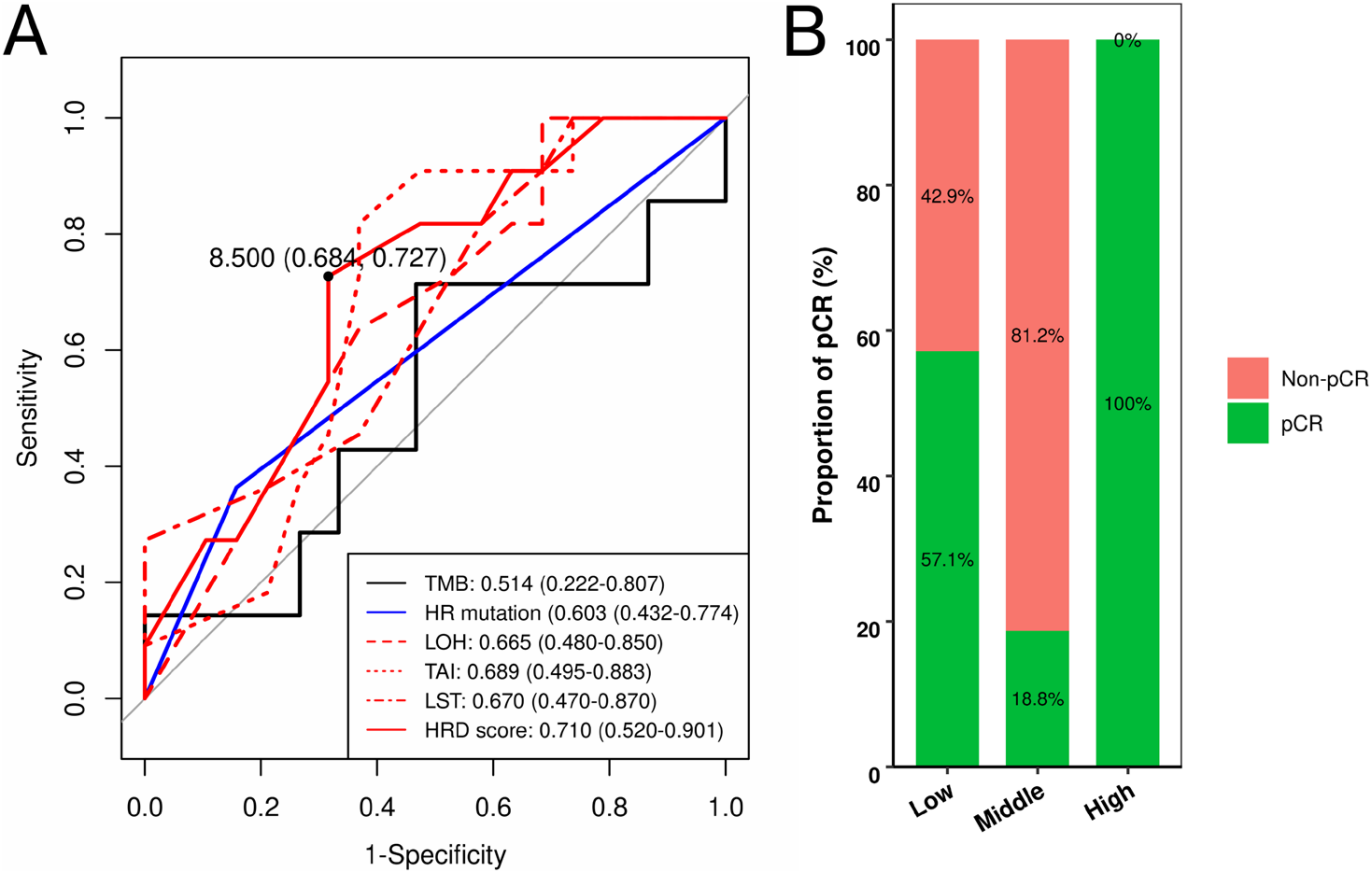
The ROC curves (*n* = 30) and stacked column charts (*n* = 32). (A) ROC curves illustrating the efficacy of TMB, HR gene mutation, and HRD score as continuous variables for discriminating pCR from non‐pCR in 30 patients. (B) Stacked column charts illustrating the proportion of patients archiving pCR among low, intermediate, and high HRD scores groups in the whole population.

A cut‐off value of 8.5 was subsequently obtained based on Youden's index. Accordingly, the 32 cases were divided into three groups based on HRD score: low (0–8), intermediate (9–37), and high (> 37). In the pCR group, the proportions of low, intermediate, and high HRD score groups were 61.54%, 23.08%, and 15.38%, respectively. In contrast, in the non‐pCR group, the proportions of low, intermediate, and high HRD score groups were 31.58%, 68.42%, and 0%, respectively (*p* = 0.017) (Figure 2B, Table S1). These results suggest that in this LARC cohort, the HRD score may have a non‐monotonic impact on the response to neoadjuvant radioimmunotherapy in terms of pCR rate. The intermediate HRD score group demonstrated the lowest pCR rate.

### 3.3 Association Between HRD Score and TIME

RSI in the high HRD score group was significantly lower than in the low HRD score group (adjusted *p* = 0.045). However, there was no significant difference in RSI between the low and intermediate HRD score groups (adjusted *p* = 1.000) (Figure S3A, Table S2). Additionally, neither the composition of TME subtypes (Fisher's exact probability, *p* = 0.988) nor the CMS classification (Fisher's exact probability, *p* = 0.966) exhibited statistical significance across the three HRD score groups (Figure S3B). These findings suggest that the previously proposed RSI and tumor subtyping systems do not explain the impact of HRD score on pCR.

To further explore the underlying mechanisms of the observed phenomena, MCPcounter and ssGSEA were used to characterize the TME across the three HRD score groups. It was found that the abundance of CD8+ T cells was numerically higher in the high HRD score group compared to the other two groups. Moreover, the abundance of CD8+ T cells in the low HRD score group was higher than in the intermediate HRD score group, although this difference only reached borderline statistical significance due to the limited sample size (adjusted *p* = 0.083) (Figure 3A,C, Table S2).

**FIGURE 3:**
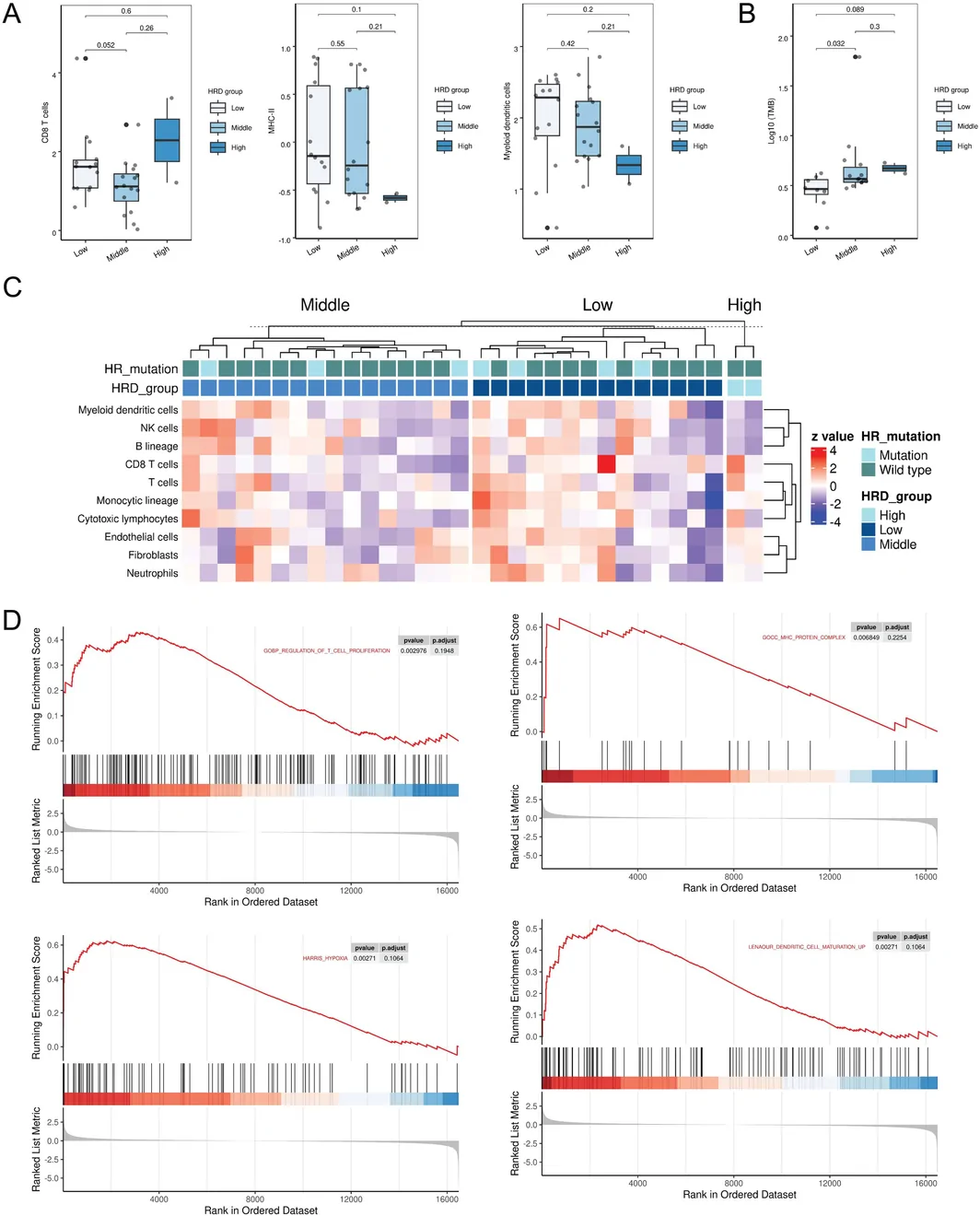
The association between HRD score and TIME. (A) Grouped boxplots showing differences in CD8+ T cells, MHC‐II molecules, and myeloid dendritic cells in the low, intermediate, and high HRD score groups. (B) Grouped boxplot demonstrating a sequential increase in TMB among the low, intermediate, and high HRD score groups. (C) A heatmap displaying the z‐transformed abundance of cells inferred from MCPcounter and enrichment score derived from single‐sample gene set enrichment analysis (ssGSEA) for 32 samples. (D) Gene Set Enrichment Analysis (GSEA) comparing the low HRD score group versus the intermediate HRD score group.

The MHC‐II molecule signature tended to be lower in the intermediate HRD score group compared to the low HRD score group. Similarly, the abundance of myeloid‐derived dendritic cells (mDCs) decreased sequentially from the low to the intermediate to the high HRD score groups in a more pronounced manner (Figure 3A,C).

In stark contrast, the TMB increased sequentially across the low, intermediate, and high HRD score groups, with a statistically significant difference observed between the low and intermediate HRD score groups (adjusted *p* = 0.050) (Figure 3B, Table S2). In addition, GSEA analysis revealed that the low HRD score group was significantly enriched in pathways related to dendritic cell (DC) maturation, MHC protein complexes, T cell proliferation, and hypoxia compared to the intermediate HRD score group (Figure 3D, Table S3).

Taken together, these results suggest that the intermediate HRD score group may exhibit a declining T cell‐inflamed TME compared to the low HRD score group. Meanwhile, the high HRD score group seems to have a higher response rate to SCRT combined with sequential chemotherapy and immunotherapy, likely due to the high intrinsic radiosensitivity of tumor cells, such as the low RSI.

## 4 Discussion

It is generally accepted that HRD status serves as an indicator of the sensitivity of tumor cells to platinum‐based chemotherapy and RT. Tumor cells with high HRD score tend to benefit more from platinum‐based chemotherapy or RT compared to those with low HRD score according to the specific cutoff value. Studies have shown that patients with BRCA1/2 mutations and HRD‐positive TNBC defined by HRD score with cutoff value 42 have significantly higher pCR rates after receiving platinum‐based neoadjuvant chemotherapy (NAC) [33]. Additionally, HR plays a critical role in the repair of DNA double‐strand breaks (DSBs), ultimately influencing response to radiation therapy to a large extent [34]. However, this study unexpectedly found that patients with HRD score between 0 and 37 did not follow the commonly adopted concept that higher HRD score is always associated with higher therapeutic efficacy. By setting appropriate cutoff value, we classified 32 samples into three groups based on their HRD score: low (0–8), intermediate (9–37) and high (> 37). Strikingly, the pCR rate was significantly higher in the low HRD score group compared to the intermediate HRD score group (57.1% vs. 18.8%). This finding challenges established perceptions and suggests that the relationship between HRD score and efficacy may be more complex in specific contexts. Further exploration into the mechanisms by which HRD influences the efficacy of neoadjuvant therapy is warranted.

Previous studies have shown that in HRD high‐grade serous ovarian cancer (HGSOC) [35], interferon (IFN)‐responsive cancer cells exhibit high expression of MHC molecules and co‐inhibitory ligands, and low expression of co‐stimulatory molecules. This leads to increased infiltration of T regulatory (Treg) and exhausted T cells (Tex) in response to HRD‐induced TMB elevation. These results suggest that HRD may affect the efficacy of neoadjuvant therapy by influencing the TIME and altering immune cell infiltration. However, in this study of pMMR LARC patients, we observed that when the HRD score was in the low‐to‐intermediate range (0–37), the abundance of DCs, MHC molecules, and CD8+ T cells in the TME decreased as the HRD score increased. This atypical infiltration pattern may influence the efficacy of neoadjuvant therapy. Recent studies have begun to reveal the interaction between HRD and the human TME [36, 37, 38]. These studies may contribute to in‐depth exploration to uncover the divergent link between HRD and the TME.

RT not only directly kills tumor cells but also induces the release of pro‐inflammatory factors that promote immune cell infiltration and remodel the TIME [39]. Previous studies have shown that prolonged RT may trigger immune dysfunction and reduce lymphocyte infiltration. However, the STELLAR and RAPIDO studies have demonstrated that neoadjuvant SCRT in LARC patients has similar efficacy to long‐course RT and avoid immune dysfunction [40, 41]. In light of these findings, the aim of this study was to explore effective biomarkers for predicting the efficacy of neoadjuvant immunotherapy in pMMR LARC patients, which typically exhibit a low immune phenotype. We implemented a treatment regimen of neoadjuvant SCRT combined with sequential chemotherapy and immunotherapy in 32 patients, expecting to activate and enhance the efficacy of immunotherapy through SCRT. However, this regimen did not achieve the desired effect in the intermediate HRD score group of patients. This may be related to the low abundance of DCs, MHC molecules, and CD8+ T‐cell infiltration in the TME prior to treatment. Based on these observations, we hypothesize that SCRT may fail to convert all pMMR LARCs into immunologically active “hot” tumors, particularly in the HRD score intermediate group, where there is a low proportion of immune cells in the TME before RT. In fact, existence of considerable amount of immune infiltration before treatment is prerequisite for improved efficacy to genotoxic therapies through cGAS‐STING pathway [42]. We also believe that the HRD score holds potential as a biomarker to identify pMMR LARC patients who may benefit from neoadjuvant RT combined with immunotherapy.

A genome‐scale analysis of somatic alterations in colorectal cancer has revealed that MSS tumors typically exhibit near‐diploid genomes and CNVs compared to MSI tumors [42]. Further research indicated that a high degree of CNVs is closely associated with decreased expression of immune cell markers, particularly a significant reduction in CD8+ T‐cell numbers, suggesting that CNVs may negatively impact the TIME [43]. Given that HRD is largely driven by CNVs, this raises an intriguing question: in samples with low to intermediate HRD score, could alterations in the tumor immune microenvironment be attributed to increasing CNVs? However, it is noteworthy that in samples with high HRD score, CNVs did not significantly suppress immune responses. This may be due to the combined effects of multiple factors, with the enhanced intrinsic sensitivity of HRD‐high tumors to nCRT potentially playing a dominant role. Additionally, the impact of CNVs on the TIME across different cancer types, as well as their correlation with HRD score, requires further validation. Future studies should continue to explore the complex interactions between these factors to provide new strategies and insights for improving neoadjuvant therapy in cancer.

There are several limitations in the present study. These preliminary results should be interpreted with caution. The comprehensive assessment of the relationship between HRD score and neoadjuvant therapy outcomes was hindered due to the limited sample size. Limited sample size also restricts the utilization of multivariate regression analysis to identify factors independently associated with pCR. The detection method used to derive HRD score was not benchmarked against other clinically approved NGS methods. The eventually derived HRD score may be different due to the genomic regions differentially covered by various NGS. Therefore, the establishment of a clinically meaningful cutoff value of HRD score was impossible in this study. Finally, a deep exploration into the mechanism by which HRD score or CNVs influence the TIME is lacking. In addition, the association of HRD score with pCR cannot be solely attributed to RT due to the subsequent usage of immunotherapy. The association of HRD score and response pattern to neoadjuvant radioimmunotherapy should be further validated with a large sample size in the future in order to facilitate clinical translation.

In conclusion, we conducted an in‐depth analysis of a cohort of 32 pMMR LARC patients, exploring the link between HRD, SCRT and the TIME in a clinical setting where all patients received a neoadjuvant radioimmunotherapy regimen. Continued investigation into the mechanisms by which HRD influences the TME in pMMR LARC is essential for realizing its potential to enhance the efficacy of immunosuppressive therapy.

## Author Contributions

**Xin Ran:** writing – original draft, writing – review and editing, investigation. **He Xiao:** writing – original draft, writing – review and editing, formal analysis. **Peng Zhou:** data curation. **Juan He:** data curation. **Mengyuan Si:** data curation. **Xiuqiong Chen:** data curation. **Xiaona Su:** resources. **Zhuo Chen:** resources. **Jia Du:** resources. **Xiaoyan Dai:** data curation. **Xiao Yang:** supervision. **Haode Shen:** software. **Zongsheng He:** supervision. **Fan Li:** methodology. **Mengxia Li:** conceptualization, funding acquisition. **Chuan Chen:** conceptualization, project administration.

## Funding

This work was supported by the Programme for Innovative Talents of Army Medical Centre of PLA [grant numbers ZXYZZKY07]; and the Clinical Medical Research Project of Army Medical Centre of PLA [grant number 2023XLC12].

## Ethics Statement

This study was approved by the Ethics Committee of the institution (Ethical Review of Medical Research [2024] No. 95). All patients signed written informed consent before sample collection.

## Consent

All patients signed written informed consent for permitting publication of their clinical information and genomic data.

## Conflicts of Interest

The authors declare no conflicts of interest.

## Supporting information


**Table S1:** Association of clinical factors and MPR or pCR in the whole population (*n* = 32).
**Table S2:** Kruskal–Wallis test for immune‐related indices among three groups categorized according to HRD score.
**Table S3:** GESA for DEG HRD score Low group vs. HRD score Middle group.

**Figure S1:** Flowchart of sequencing and analysis after taking biopsies of 32 samples.

**Figure S2:** Difference of HRD score between pathological response, as well as its predictive value for PCR in the whole population. (A) Grouped boxplots of HRD score between pCR and non‐pCR patient (*n* = 32). (B) ROC curves demonstrating the predictive performance of TMB, HR gene mutation, and HRD score in distinguishing between the pCR and non‐pCR.

**Figure S3:** TIME features among HRD score groups. (A) Grouped boxplot illustrating the differences in the radiosensitivity index (RSI) across low, intermediate, and high HRD score groups. (B) Stacked column charts showing the proportions of the four consensus molecular subtypes and four immune subtypes across low, intermediate, and high HRD score groups.

## Data Availability

The raw sequence data used for inferring the HRD score reported in this paper have been deposited in the Genome Sequence Archive at the National Genomics Data Center (Beijing China) under BioProject PRJCA035982.
