# Supplementary material for: Association of HRD Score With Efficacy of Neoadjuvant Short‐Course Radiotherapy Plus Sequential Chemoimmunotherapy in pMMR Locally Advanced Rectal Cancer
Source: Cancer Rep (Hoboken). 2026 Oct 1;9(10):e70708. doi: 10.1002/cnr2.70708 (PMC13630485; doi:10.1002/cnr2.70708)
Supplement: Supplementary file 1 [file CNR2-9-e70708-s002.pdf]

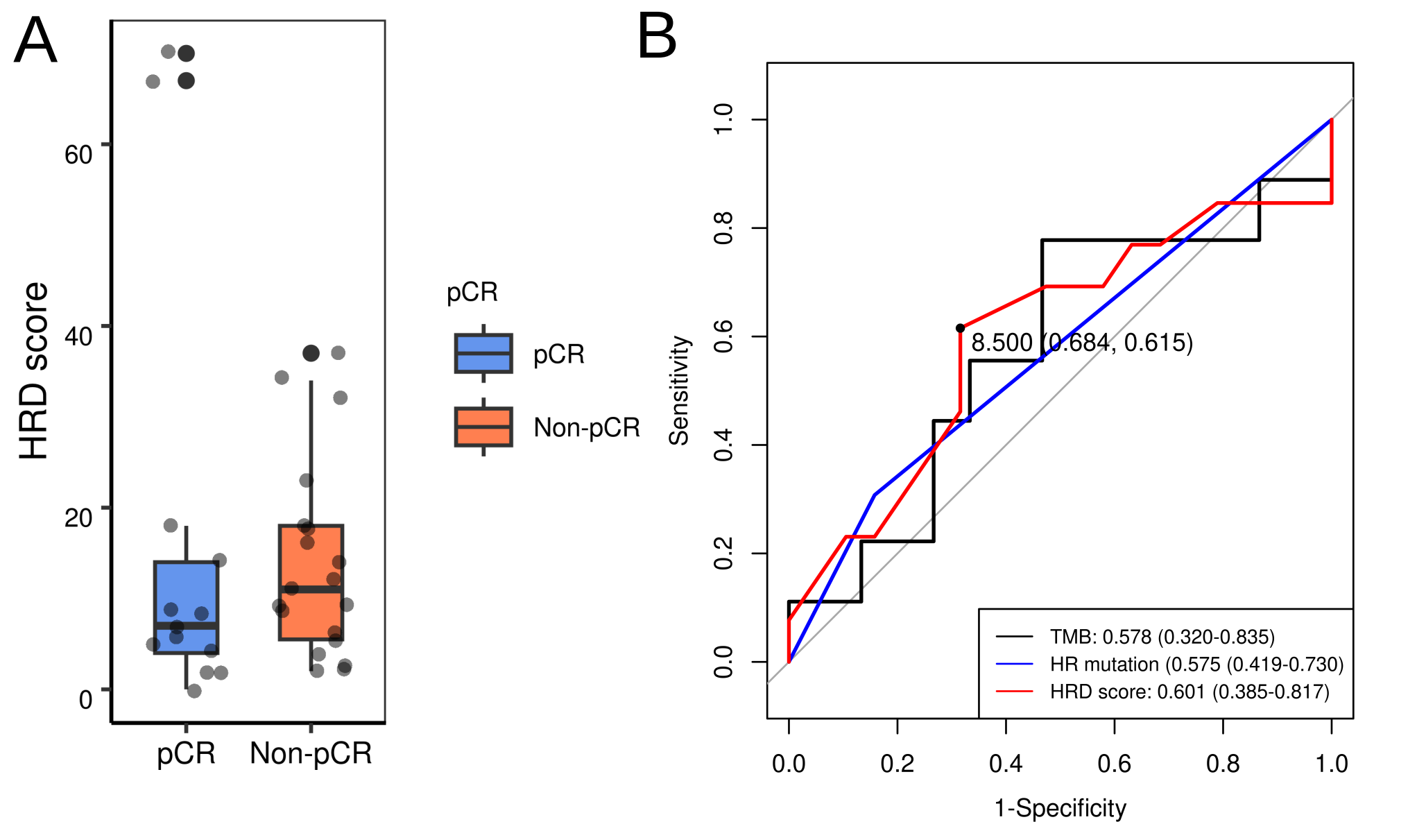
A
HRD score
0
20
40
60
pCR
Non-pCR
pCR
pCR
Non-pCR
B
Sensitivity
1-Specificity
0.0
0.2
0.4
0.6
0.8
1.0
8.500 (0.684, 0.615)
TMB: 0.578 (0.320-0.835)
HR mutation (0.575 (0.419-0.730)
HRD score: 0.601 (0.385-0.817)
